# Marine hydrocarbonoclastic bacteria as whole-cell biosensors for *n*-alkanes

**DOI:** 10.1111/1751-7915.12286

**Published:** 2015-04-15

**Authors:** Emma Sevilla, Luis Yuste, Fernando Rojo

**Affiliations:** Departamento de Biotecnología Microbiana, Centro Nacional de Biotecnología, CSICDarwin 3, Cantoblanco, Madrid, 28049, Spain

## Abstract

Whole-cell biosensors offer potentially useful, cost-effective systems for the *in-situ* monitoring of seawater for hydrocarbons derived from accidental spills. The present work compares the performance of a biosensor system for the detection of alkanes in seawater, hosted in either *Escherichia coli* (commonly employed in whole-cell biosensors but not optimized for alkane assimilation) or different marine bacteria specialized in assimilating alkanes. The sensor system was based on the *Pseudomonas putida* AlkS regulatory protein and the *PalkB* promoter fused to a gene encoding the green fluorescent protein. While the *E. coli* sensor provided the fastest response to pure alkanes (25-fold induction after 2 h under the conditions used), a sensor based on *Alcanivorax borkumensis* was slower, requiring 3–4 h to reach similar induction values. However, the *A. borkumensis* sensor showed a fourfold lower detection threshold for octane (0.5 μM), and was also better at sensing the alkanes present in petrol. At petrol concentrations of 0.0125%, the *A. borkumensis* sensor rendered a sevenfold induction, while *E. coli* sensor showed no response. We discuss possible explanations to this behaviour in terms of the cellular adaptations to alkane uptake and the basal fluorescence produced by each bacterial strain, which was lowest for *A. borkumensis*.

## Introduction

Marine environments face frequent challenges from spills of crude oil or its derivatives, the consequence of accidents that occur during their offshore extraction, transportation or use. Very large-scale accidents, such as those involving the Exxon-Valdez and Prestige oil tankers, or the recent, prolonged spill that occurred following the explosion of the Deepwater Horizon oil platform, are fortunately infrequent, but when they do occur their ecological and economic consequences can be severe (Bragg *et al*., 1994; Peterson *et al*., 2003; Albaiges *et al*., 2006; Bernabeu *et al*., 2009; Lubchenco *et al*., 2012; Ryerson *et al*., 2012). Small-scale spills of crude oil and fuels, which are much more common, have a cumulative effect that can also deteriorate aquatic and marine ecosystems. Efficient, cost-effective systems that allow the *in-situ* monitoring of such environments for hydrocarbon contaminants derived from crude oil are therefore required (Kalogerakis *et al*., 2015). Gas chromatography coupled to mass spectrometry (GC-MS) and high-pressure liquid chromatography (HPLC) are the main analytical methods used to test for the presence of hydrocarbons in water samples. Though accurate, these techniques require heavy, complicated and expensive equipment. Biosensors might provide an easy-to-use, fast and cost-effective complementary tool that, while not as accurate as the latter methods, could be very useful for the rapid detection of hydrocarbons in water (Diplock *et al*., 2010; van der Meer and Belkin, 2010). Biosensors are analytical devices based on biological components, such as whole cells or enzymes that sense a signal and generate a detectable and quantifiable response. An important difference between biosensors and techniques such as GC-MS is that the former respond to the bioavailable fraction of a pollutant, i.e. that available to the cells or to the enzyme used as the sensing device. In contrast, the traditional methods detect the total amount of solvent-extractable pollutant in a sample. Part of this, however, might be unavailable to cells, for example if the polluting molecules are adsorbed onto solids. Differentiating the bioavailable from the non-bioavailable fractions of a contaminant is important when trying to determine its ecotoxicity (Tecon and van der Meer, 2008; Tecon *et al*., 2009).

Several whole-cell biosensors have been reported that respond to different hydrocarbons (Sticher *et al*., 1997; Alkasrawi *et al*., 1999; Stiner and Halverson, 2002; Phoenix *et al*., 2003; Tecon *et al*., 2006; 2009; Kumari *et al*., 2011; Reed *et al*., 2012; Zhang *et al*., 2012). Although they perform well under laboratory conditions, these (and others) suffer a number of problems that limit their use (van der Meer *et al*., 2004; Harms *et al*., 2006). For example, the detection of hydrocarbons in water samples is particularly challenging given their low solubility in this medium; this leads to mass transfer problems that limit the availability of hydrocarbons to the cell (Bosma *et al*., 1997; Jaspers *et al*., 2001). This can substantially increase the response time of the biosensor and can lead to low signal to noise ratios. One way of improving the reliability of biosensors may be to use as hosts for the sensing systems cells that are adapted for the uptake and metabolism of hydrocarbons. However, a systematic comparison of the performance of a sensor system for hydrocarbons in different bacterial hosts has not been reported to our knowledge.

In recent years, a number of marine bacteria have been described that can efficiently colonize seawater contaminated with hydrocarbons, and that use many hydrocarbons as carbon sources. These bacteria may therefore play an important role in attenuating pollution (Harayama *et al*., 2004; Head *et al*., 2006). While some of these are generalist bacteria with versatile metabolisms, many others are hydrocarbonoclastic and highly specialized in the assimilation of hydrocarbons; indeed, some can use very few other compounds as carbon sources (Yakimov *et al*., 2007). These obligate oil-degrading bacteria are thought to have evolved highly efficient systems for gaining access to hydrocarbons; in fact, they normally produce surfactants and have hydrophobic outer envelopes that optimize access to the oil phase.

The present work compares the performance of a biosensor system for the detection of alkanes in seawater, hosted in either *Escherichia coli* (commonly used in many whole-cell biosensors but not optimized for alkane degradation) or different marine hydrocarbonoclastic bacteria specialized in assimilating linear alkanes. A biosensor based on hydrocarbonoclastic bacteria *Alcanivorax borkumensis* proved to be better than the equivalent biosensors based on the other strains tested, particularly when challenged to low concentrations of pure alkanes or of petrol.

## Results

### Construction of a set of reporter strains containing plasmid *pKSB1*

**Fig 1:**
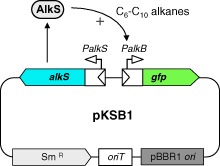
Diagram of the pKSB1 reporter plasmid. This plasmid is based on vector pSEVA431 (Silva-Rocha *et al*., 2013), which contains the replication origin of the broad-host-range pBBR1, a streptomycin resistance determinant and a transfer origin (*oriT*) to facilitate conjugation in the presence of a helper plasmid providing a conjugative apparatus. The gene coding for the AlkS transcriptional activator from the *P. putida* OCT plasmid and a *PalkB-gfp* transcriptional fusion, were introduced into the multi-cloning site of the vector. AlkS can activate transcription from the *PalkB* promoter in the presence of C_6_-C_10_ alkanes (Sticher *et al*., 1997; Panke *et al*., 1999).

The core of the sensing device used in this work was the AlkS transcriptional activator from the *Pseudomonas putida* OCT plasmid, and the AlkS-responsive *PalkB* promoter fused to the gene coding for the green fluorescent protein (GFP). The AlkS protein activates transcription from promoter *PalkB* in the presence of alkanes with 6–10 carbon atoms (Grund *et al*., 1975; Sticher *et al*., 1997; Panke *et al*., 1999). Promoter *PalkB* was fused to a variant of the *gfp* gene designed for prokaryotic transcriptional fusions and which contains the S65T ‘red shift’ and F64L ‘protein solubility’ mutations (Miller and Lindow, 1997). The *alkS* gene and the *PalkB-gfp* fusion were cloned into the broad-host-range plasmid pSEVA431 (Silva-Rocha *et al*., 2013), thus obtaining plasmid pKSB1 (see Fig. 1). Plasmid pSEVA431 bears the origin of replication of the broad-host-range plasmid pBBR1, and was selected since it was found to replicate in all the bacterial strains examined in the present work. Other vectors based on the replication origin of the broad-host-range plasmid RK2 did not replicate in any of the marine strains tested.

The reporter plasmid pKSB1 was transferred to *E. coli* W3110 (Jishage and Ishihama, 1997) and to the marine hydrocarbonoclastic bacteria *Marinobacter hydrocarbonoclasticus* VT8 (Gauthier *et al*., 1992), *A. borkumensis* SK2 (Yakimov *et al*., 1998), *Thalassolituus oleivorans* Mil-1 (Yakimov *et al*., 2004), *Oleiphilus messinensis* ME102 (Golyshin *et al*., 2002) and *Cycloclasticus sp*. ME7 (a Mediterranean variant of *Cycloclasticus pugetii*; Dyksterhouse *et al*., 1995). The plasmid was successfully mobilized from *E. coli* to the marine bacteria by conjugation, except for *Cycloclasticus sp*. ME7, for which electroporation was required (see *Experimental procedures*). The plasmid was able to replicate in all the bacteria tested. Growth of the *Oleiphilus* strain was however very slow both in the absence or presence of the plasmid, and only very low turbidity values were reached. The marine strains were cultivated in the artificial seawater medium ONR7a (Dyksterhouse *et al*., 1995), while *E. coli* was routinely propagated in M9 mineral salts medium (Sambrook and Russell, 2001). Cultivation of *E. coli* in seawater ONR7a was only possible after a previous acclimatization period, although growth speed decreased to about a half of that detected in M9 medium. Table 1 summarizes the growth conditions and characteristics of the strains used as hosts for the biosensor system.

**Table 1:** Characteristics of the strains used as hosts for the alkane biosensor

Strain^a^	*Marinobacter*	*Alcanivorax*	*Thalassolituus*	*Oleiphilus*	*Cycloclasticus*	*E. coli*
Growth medium	ONR7a	ONR7a	ONR7a	ONR7a	ONR7a	M9
Carbon source^b^(for growth)	1% Pyruvate	1% Pyruvate	2.5% C_14_	2.5% C_14_	0.25% Naphthalene	0.4% Glucose
Carbon source^b^ (conjugation assays)	C_16_ vapours	C_14_ vapours	C_14_ vapours	C_14_ vapours	–	–
Carbon source^b^ (reporter assays)	None	1% Pyruvate	1% Acetate	1% Tween-20	0.25% Naphthalene	0.4% Glucose
Growth temperature	30°C	30°C	25°C	25°C	25°C	37°C
Doubling time	78 ± 2 min	198 ± 8 min	1445 ± 11 min	ND^e^	211 ± 5 min	73 ± 1 min
MIC (solid)^c^	32 μg/ml	16 μg/ml	2 μg/ml	1 μg/ml	12 μg/ml	1.5 μg/ml
MIC (liquid)^c^	32 μg/ml	32 μg/ml	3 μg/ml	1 μg/ml	24 μg/ml	1 μg/ml
Sm^r^ mutants^d^	< 10^−6^	< 10^−6^	< 10^−6^	< 10^−5^	< 10^−6^	< 10^−6^

a The strains analysed were *M. hydrocarbonoclasticus* VT8, *A. borkumensis* SK2, *T. oleivorans* Mil-1, *O. messinensis* ME102, *Cycloclasticus* sp. ME7 and *E. coli* W3110.

b Percentage carbon sources, given as v/v for C_14_ and Tween-20, and as w/v for all other compounds.

c Minimum inhibitory concentration for streptomycin in solid (agar plates) or liquid media.

d Number of spontaneous mutants resistant to streptomycin appearing on agar plates after 15 days.

e ND, not-determined. *Oleiphilus* reached a maximum turbidity (A_600_) of 0.2 when cultivated in liquid medium with C_14_ as the carbon source.

### Time-dependent response of the reporter strains to different alkanes

**Fig 2:**
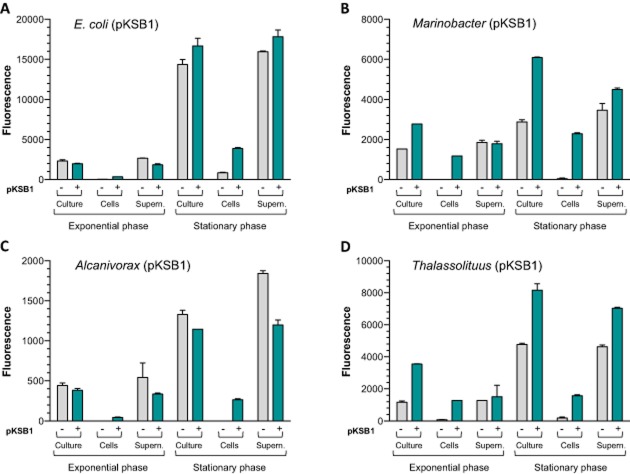
Background fluorescence of the biosensor strains. Bacterial strains *E. coli* W3110 (A), *M. hydrocarbonoclasticus* VT8 (B), *A. borkumensis* SK2 (C) and *T. oleivorans* Mil-1 (D), containing or lacking the reporter plasmid pKSB1 (indicated as + or –, respectively) were cultivated either in mineral salts M9 medium with glucose (*E. coli*) or in ONR7a sea water medium containing as carbon source pyruvate (*M. hydrocarbonoclasticus* and *A. borkumensis* SK2) or acetate (*T. oleivorans* Mil-1). At mid-exponential phase (A_600_ of 0.5), or at the stationary phase of growth (A_600_ of 1 except for *E. coli*, that reached an A_600_ of 3), samples were collected and the fluorescence emitted (excitation at 480 nm, emission at 520 nm) was measured using either the culture sample (indicated as ‘culture’), the cells collected after centrifugation and re-suspended in the same volume of fresh medium (indicated as ‘cells’), or the cell-free culture supernatant (indicated as ‘supernatant’). Note that the values corresponding to *E. coli* in stationary phase correspond to samples that contained three times more cells than those of the other bacterial strains, since cultures grew to a higher turbidity. The values shown represent the average of three independent experiments; the standard deviation is indicated.

Preliminary assays showed that, when cultivated in the absence of alkanes, the stationary phase cultures of all the strains analysed showed significant fluorescence at the excitation/emission wavelengths characteristic of the GFP protein, which could compromise the reporter assays. To analyse whether background fluorescence was due to a basal expression of the *gfp* gene in the absence of alkanes, or to compounds produced and perhaps secreted by the cells, fluorescence measurements were made with cells containing or lacking the reporter plasmid pKSB1, cultivated in their appropriate minimal salts medium (see legend to Fig. 2) and collected at mid-exponential exponential phase (A_600_ of 0.5) or in stationary phase (A_600_ of 1 except for *E. coli*, that reached an A_600_ of 3). To determine whether fluorescence was due to compounds expelled to the medium, cells were spun down and re-suspended in fresh medium and fluorescence measured both in the re-suspended cells and in the supernatant. As shown in Fig. 2, all strains showed significant background fluorescence, particularly in stationary phase cultures, irrespective of the absence or presence of the reporter plasmid in the cells. In *E. coli*, this fluorescence was much higher in the culture supernatants than in the cells, suggesting that it may derive to a large extent from compounds released to the culture medium. The presence of the reporter plasmid increased the fluorescence emitted by the cells by fourfold, although fluorescence was much lower than that observed in the culture supernatants, suggesting that there is some basal expression of GFP in the absence of alkanes, although its contribution to the overall fluorescence is less than 25%. The cells of *A. borkumensis* showed the lowest background fluorescence, and most of it derived from compounds present in the culture supernatants. The values observed for *M. hydrocarbonoclasticus* and *T. oleivorans* were in most cases midway between those of *E. coli* and *A. borkumensis*. The contribution of the basal expression of GFP to the overall background fluorescence was substantial in both *M. hydrocarbonoclasticus* and *T. oleivorans*, although a significant fluorescence was also detected in culture supernatants irrespective of the presence or absence of the reporter plasmid.

To minimize the problem of background fluorescence in the bioreporter assays, these were performed with cells that were previously centrifuged and re-suspended in fresh medium containing the appropriate carbon source for each strain (glucose, pyruvate or acetate, see Table 1). However, the *Marinobacter* strain quickly developed a strong fluorescence signal once again, despite there being no hydrocarbons present that could induce GFP production. This was reduced if the fluorescence assays were performed with the *Marinobacter* cells re-suspended in a fresh medium containing no carbon source.

**Fig 3:**
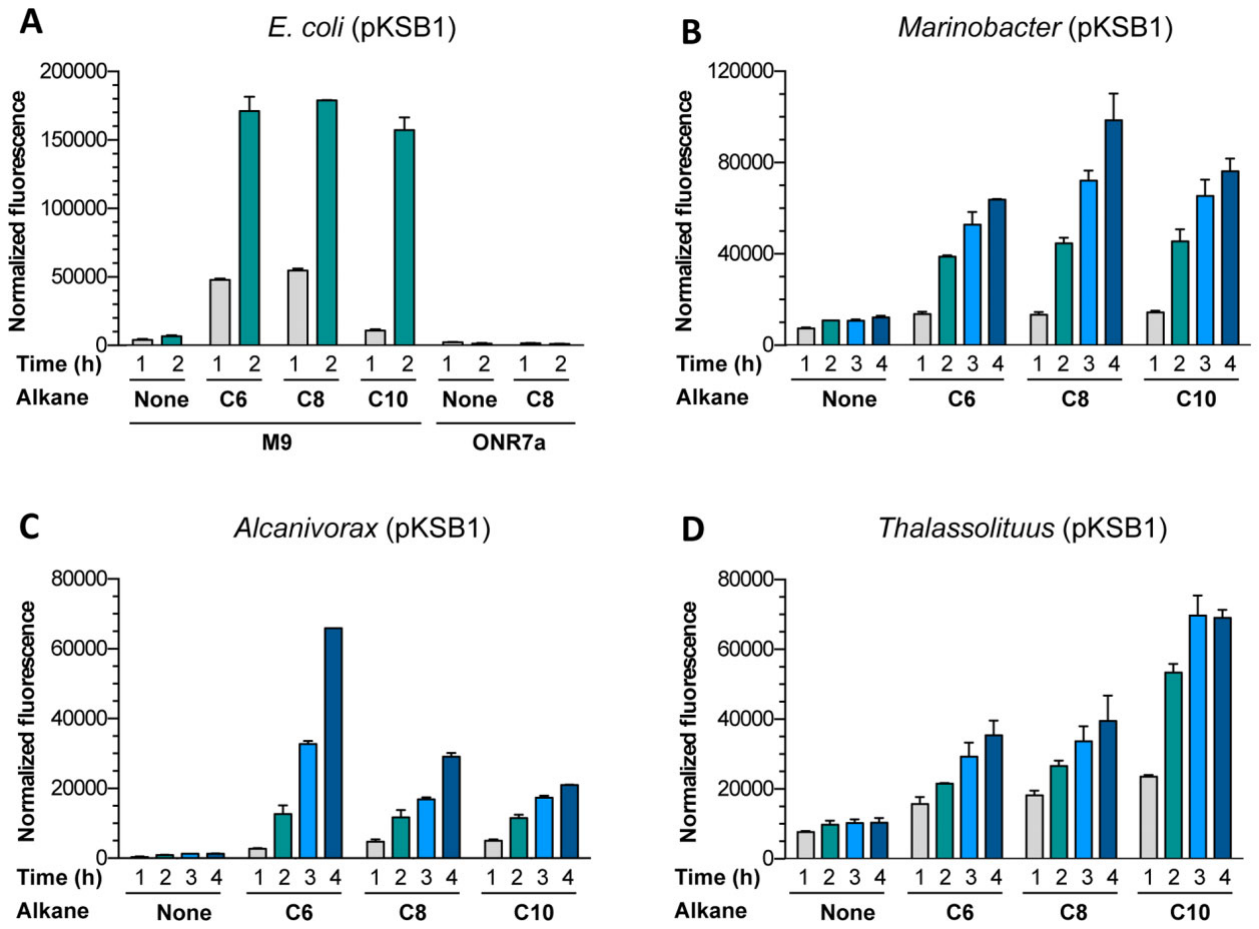
Time-dependent response of the reporter strains to C_6_, C_8_ and C_10_ alkanes. Cells of the different reporter strains were incubated in the absence or presence of C_6_, C_8_ or C_10_ alkanes at 1% (v/v) for different time periods (1 to 4 h) as indicated in *Experimental procedures*. *Escherichia coli cells* were cultivated in M9 mineral medium and confronted to samples containing or lacking alkanes, prepared either in M9 medium or in ONR7a medium. The other three strains were cultivated in ONR7a medium, and confronted to samples with or without alkanes, prepared in ONR7a. The figure shows the fluorescence response of (A) *E. coli* W3110 (pKSB1); (B) *M. hydrocarbonoclasticus* VT8 (pKSB1); (C) *A. borkumensis* SK2 (pKSB1); (D) *T. oleivorans* Mil-1 (pKSB1). The values shown correspond to normalized fluorescence and represent the average of three independent experiments; the standard deviation is indicated.

Different pure *n*-alkanes (hexane, octane, decane, dodecane, tetradecane, hexadecane, octadecane and eicosane, abbreviated hereafter as C_6_, C_8_, C_10_, C_12_, C_14_, C_16_, C_18_ and C_20_, respectively) were added to the cells at a concentration of 1%, which is above their solubility limit in water. A control assay involving no alkane was performed in parallel. An alkane-only control, lacking cells, was also included. The samples were incubated at 30°C with agitation in closed glass vials limiting the headspace to 10% of the total volume. At different times, the fluorescence was measured as indicated in *Experimental procedures*, normalizing the values observed relative to the culture turbidity. In the absence of *n*-alkanes, the normalized fluorescence observed after 2 h for the reporter strains containing plasmid pKSB1 was lowest for *A. borkumensis* (920 ± 60 units), intermediate for *E. coli* (6770 ± 715 units), and significantly higher for the rest of the strains (9710 ± 1180 units for *T. oleivorans*, 9715 ± 120 units for *O. messinensis*, 10530 ± 385 units for *Cycloclasticus sp*. ME7 and 10860 ± 85 units for *M. hydrocarbonoclasticus*). As shown in Fig. 3, the *E. coli*, *Marinobacter*, *Alcanivorax* and *Thalassolituus* strains containing plasmid pKSB1 showed a good response to C_6_, C_8_ and C_10_ alkanes. The response was already clear after 1 h, and increased steadily over time. No response was obtained with alkanes of 11 or more carbon atoms. This agrees with previous reports that, at least in *E. coli*, the AlkS/*PalkB* system responds only to C_6_–C_10_ alkanes (Sticher *et al*., 1997; Reed *et al*., 2012). The *E. coli* strain responded faster than the others; in fact the assay was stopped after 2 h since at longer incubation times the fluorescence signal saturated the detector. If the background signal obtained in the absence of alkanes was subtracted to that obtained in the presence of alkanes, the response of the *E. coli* biosensor after a 2 h incubation was about fivefold higher than that of the other reporter strains. However, allowing the bioreporter assay to proceed for 4 h led to a clear improvement in the response of the *Marinobacer*, *Alcanivorax* and *Thalasolituus* strains, with little increase in the background signal (Fig. 3). The *E. coli* cells seemed to be sensitive to the ionic strength of the incubation medium, since the alkane response was very good when cells were incubated in the M9 mineral salts medium but became undetectable when the assay was performed in the artificial seawater medium ONR7a, which has a much higher ionic strength (Fig. 3A). The sensitivity of *E. coli* bioreporters to seawater has been observed previously (Tecon *et al*., 2010); these authors indicated that seawater samples had to be diluted at least four times to avoid inhibition of the reporter assays by the salt. As an alternative approach, prior to the reporter assays *E. coli* cells were cultivated directly in ONR7a medium, conditions in which growth is possible after an acclimatization period, although growth rate decreases significantly. However, the response to alkanes of *E. coli* cells adapted to grow in ONR7a medium was about four to sixfold lower than that of cells cultivated in M9 medium, and the variability of the assays was higher (not shown).

The *Oleiphilus* and *Cycloclasticus* strains were not useful as biosensors since their response to alkanes after 4 h was very poor; indeed, up to 4 days were needed for any significant fluorescence to be seen (not shown). These strains were therefore discarded from further analyses.

**Fig 4:**
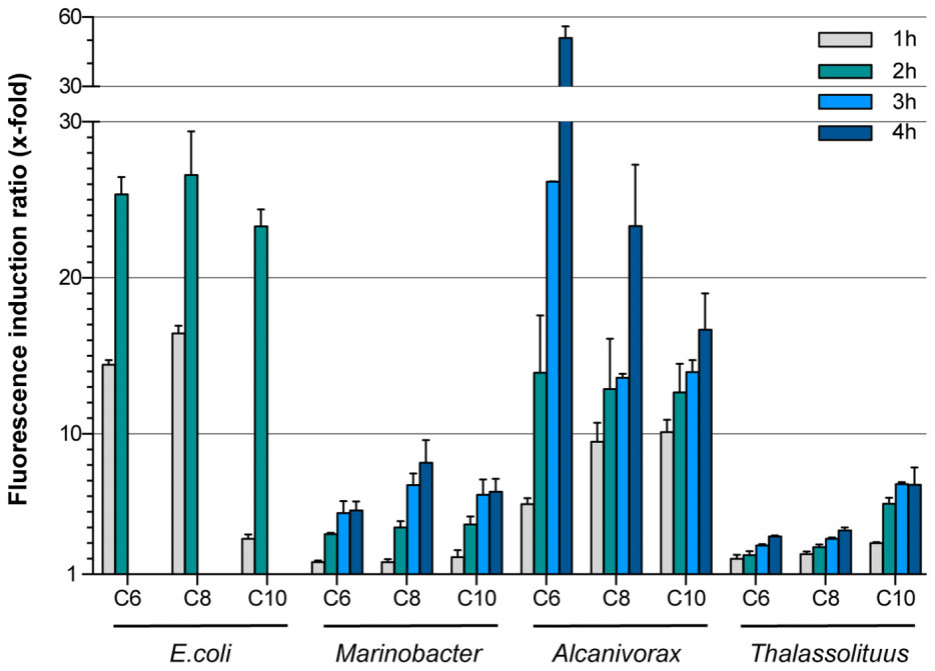
Fluorescence–induction ratios in response to C_6_, C_8_ or C_10_ alkanes for different reporter strains. Cells of *E. coli* W3110 (pKSB1), *M. hydrocarbonoclasticus* VT8 (pKSB1), *A. borkumensis* SK2 (pKSB1) or *T. oleivorans* Mil-1 (pKSB1) were incubated in the absence or presence of C_6_, C_8_ or C_10_ alkanes at 1% (v/v) for 1–4 h. Assays were performed either in M9 minimal medium (for *E. coli* reporter) or in ONR7a medium (for *Marinobacter*, *Alcanivorax* and *Thalassolituus* reporter strains). The graph shows the fluorescence induction ratios, calculated as the normalized fluorescence observed in alkane-containing samples over that of control samples lacking alkanes. Values correspond to the average of three independent experiments; the standard deviation is indicated.

When considering the fluorescence induction ratios (signal observed in the presence of alkanes divided by that observed in its absence), the background fluorescence in the absence of alkanes had an important impact. Induction of fluorescence was strong in the *Alcanivorax* (around 50-fold that seen in the absence of alkanes for C_6_ after 4 h) and *E. coli* (around 25-fold after 2 h) strains, and clearly lower (six to eightfold) in the *Marinobacter* and *Thalassolituus* strains (Fig. 4). The *E. coli* and *Marinobacter* strains showed a similar response, irrespective of the chain length of the alkanes, while the *Alcanivorax* strain responded better to C_6_ than to C_10_. The *Thalassolituus* strain showed the opposite behaviour (Figs. 3 and 4). This suggests differences in the way in which each of these bacterial strains gains access to the different alkanes.

### Detection threshold of the reporter strains to octane

**Fig 5:**
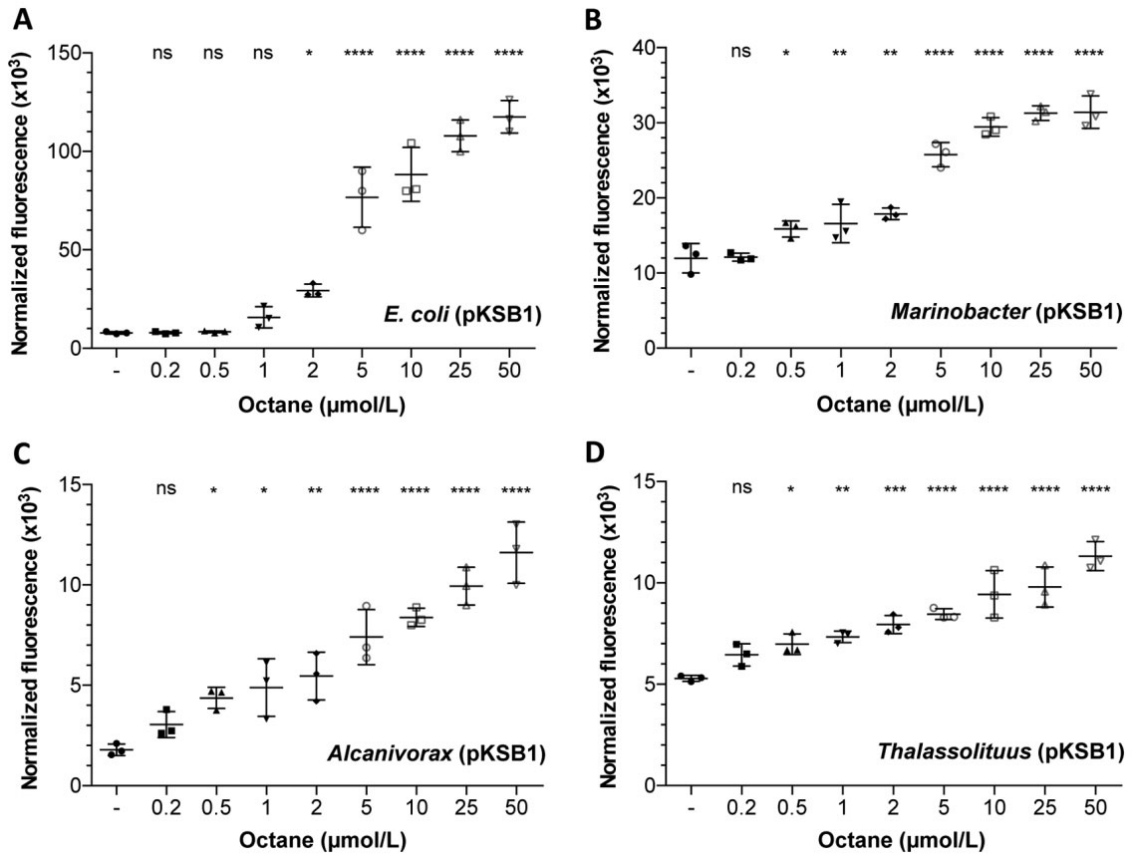
Detection threshold of the reporter strains. Cells of (A) *E. coli* W3110 (pKSB1), (B) *M. hydrocarbonoclasticus* VT8 (pKSB1), (C) *A. borkumensis* SK2 (pKSB1) or (D) *T. oleivorans* Mil-1 (pKSB1), were incubated for 2 h in the absence or presence of the indicated concentrations of octane (added from stock solutions prepared in dimethyl sulphoxide). Assays were performed either in M9 mineral medium (for *E. coli* reporter) or in ONR7a medium (for *Marinobacter*, *Alcanivorax* and *Thalassolituus* reporter strains). The normalized fluorescence observed is indicated. Values represent the mean and the standard deviation of three biological replicates. Asterisks indicate significant differences between octane-treated and non-treated samples (one-way ANOVA; *P < 0.1; **P < 0.01; ***P < 0.001; ****P < 0.0001; ‘ns’ no significant difference).

**Fig 6:**
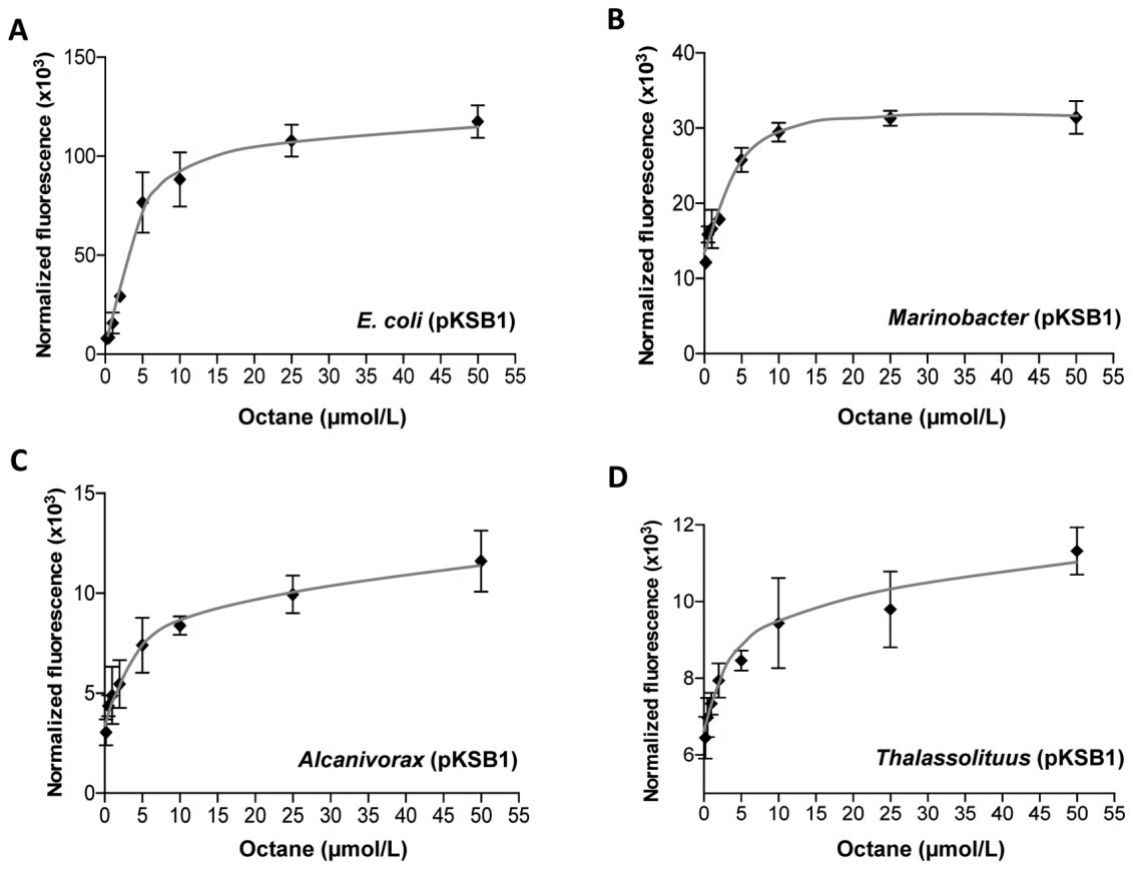
Intensity of the response of the reporter strains as a function of octane concentration. (A) *E. coli* W3110 (pKSB1), (B) *M. hydrocarbonoclasticus* VT8 (pKSB1), (C) *A. borkumensis* SK2 (pKSB1), (D) *T. oleivorans* Mil-1 (pKSB1). Values derive from those presented in Fig. 5.

The sensitivity and linearity of the reporter strains' response to octane were determined by fluorescence assays in the presence of increasing alkane concentrations (0.2 μmol/L, 0.5 μmol/L, 1 μmol/L, 2 μmol/L, 5 μmol/L, 10 μmol/L, 25 μmol/L and 50 μmol/L). All assays were conducted for 2 h in triplicate, and the data analysed by one-way analysis of variance. The *Marinobacter*, *Alcanivorax* and *Thalassolituus* strains were able to detect octane concentrations as low as 0.5 μM with statistical significance (Fig. 5B–D). However, the *E. coli* strain did not respond to octane concentrations lower than 2 μM (Fig. 5A). For the *E. coli, Marinobacter* and *Alcanivorax* strains, the response was linear up to 5 μM octane (R^2^ > 0.99); at higher octane concentrations the signal increase declined, and little or no further increase was observed above 10 μmol/L octane (Fig. 6). This is consistent with the water solubility limit of this hydrocarbon (about 6.3 μM). For the *Thalassolituus* strain, loss of linearity started at octane concentrations of 2.5 μM.

### Response of reporter strains to water contaminated with petrol or crude oil

**Fig 7:**
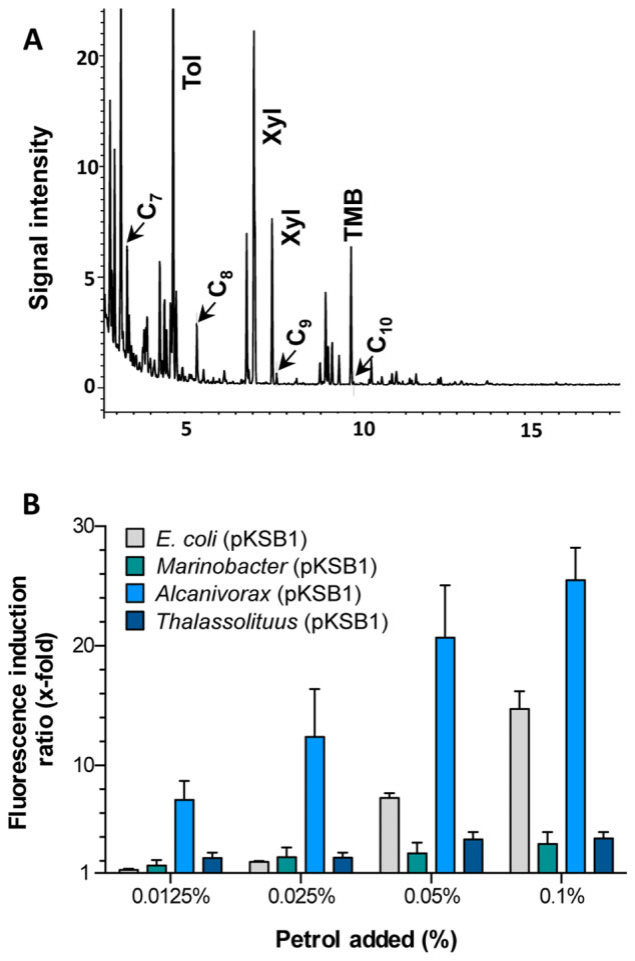
Response of reporter strains to commercial petrol. (A) GC-MS analysis of the petrol used, indicating peaks corresponding to C_7_, C_8_, C_9_ and C_10_ alkanes (the latter two alkanes rendered very small peaks). Those corresponding to toluene (Tol), xylene (Xyl; it was not possible to differentiate *m*-xylene from *o*-xylene) and trimethyl-benzene (TMB), are also indicated. (B) Fluorescence induction ratio of reporter strains after 2 h in the presence of different concentrations of petrol added to M9 minimal medium (for *E. coli*) or ONR7a (for the *Marinobacter*, *Alcanivorax* and *Thalassolituus* reporter strains). Values are the average of three independent assays; the standard deviation is indicated.

Due to their hydrophobicity, alkanes have a strong tendency to partition into organic solutes such as petrol (gasoline) or crude oil. This could reduce the efficiency of biosensors designed to detect hydrocarbons in contaminated seawater. The behaviour of the present biosensor strains when confronted with water containing petrol or crude oil was investigated in the same way as for the pure alkanes. The petrol used, obtained from a commercial filling station, contained significant amounts of C_7_ (121 mM) and C_8_ (38 mM) alkanes, and smaller amounts of C_9_ (5.2 mM) and C_10_ (0.9 mM) alkanes (Fig. 7A). Toluene, xylene and trimethylbenzene were abundant. The total linear short-chain alkanes in the petrol reached a concentration of 165 mM. However, when added to the water samples in the bioreporter assays, the petrol was diluted by more than three orders of magnitude, bringing the *n*-alkane concentration into the micromolar range. Different final concentrations of petrol (0.1%, 0.05%, 0.025% and 0.0125% v/v) were added directly to the M9 mineral salts medium for the *E. coli* strain, or the ONR7a medium for the *Marinobacter*, *Alcanivorax* and *Thalassolituus* strains. The sample was vortexed to ensure mixing, and then added to the medium containing the reporter strains. A clear fluorescence signal was detected after 2 h for all four strains (Fig. 7B). At the highest concentration of petrol used, the response of the *Alcanivorax* reporter almost doubled that of the *E. coli* strain, while the response of the *Marinobacter* and *Thalassolituus* strains was much poorer, in part due to the high fluorescence recorded in the absence of the inducer, which reduced the final fluorescence induction ratio. When added at 0.05%, the *Alcanivorax* strain returned induction values about three to four times higher than those of the other strains. At petrol concentrations of 0.025% or lower, only the *Alcanivorax* strain rendered a significant response (Fig. 7B).

**Fig 8:**
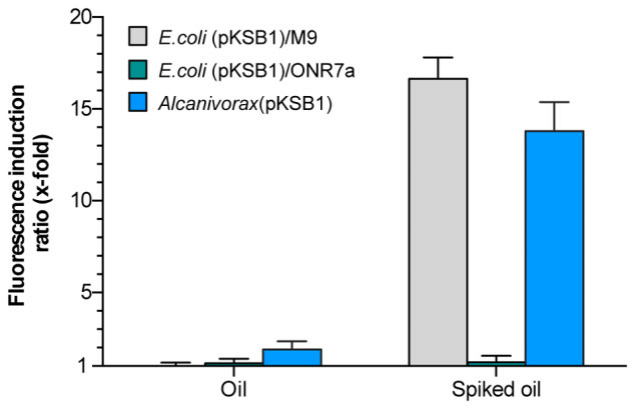
Response of reporter strains to crude oil. Dansk blend crude oil, spiked or not with a mixture of C_6_-C_14_ *n*-alkanes, was directly added to the reporter assays at a final concentration of 0.1%. Assays were performed with samples prepared either in M9 mineral salts medium, or in ONR7a medium, as indicated. A control sample to which no oil was added was assayed in parallel. The ratio of the fluorescence observed after 2 h in the presence of the oil, relative to the control sample, is indicated. Values correspond to the average of three independent assays; the standard deviation is indicated.

The response of the *Alcanivorax* and *E. coli* reporter strains to crude oil was also analysed. For this, a Dansk blend crude oil (a mixture of crude oils from multiple fields in the Danish sector of the North Sea) was used. Gas chromatography coupled to mass spectrometry chromatography analysis of this oil blend showed it to have very low or undetectable amounts of *n*-alkanes shorter than 12 carbon atoms (data not shown). Crude oil was added directly to the fluorescence assays at a final concentration of 0.1% (v/v). As predicted from the lack of C_6_-C_10_ *n*-alkanes in the oil, neither the *E. coli* nor the *Alcanivorax* reporter strains showed any clear response. However, when the oil was previously spiked with a mixture of C_6_-C_14_ *n*-alkanes (7% each, final concentration), a clear response was observed for both strains, the fluorescence induction values being close to 15-fold in both cases (Fig. 8). This is consistent with the high amount of alkanes added to the crude oil. The *E. coli* strain showed a good fluorescence response only if the assay was performed in M9 mineral salts medium; no signal was seen when ONR7a medium was used (Fig. 8).

## Discussion

The aim of the present work was to compare the performance of a biosensor system for *n*-alkanes when introduced into different bacterial strains highly – or not at all – specialized in the degradation alkanes. The hope was that the specialized alkane degraders, which might be expected to have optimized systems for the uptake of *n*-alkanes, would react faster and more efficiently than non-specialized bacteria to the presence of these compounds at low concentration in seawater samples. The eventual goal was to tackle one of the limitations of currently available biosensors for hydrocarbons, namely the low mass transfer of the hydrocarbon from the water phase to the sensing cells. This is believed to reduce the sensitivity and speed of response of these analytical devices (Bosma *et al*., 1997; van der Meer *et al*., 2004; Tecon and van der Meer, 2008; Diplock *et al*., 2010). The biosensor system used was based on the *n*-alkane-responsive AlkS transcriptional activator from the *P. putida* OCT plasmid and the fusion of the AlkS-activated *PalkB* promoter to the *gfp* gene.

All strains showed detectable background fluorescence in the absence of alkanes, even if cells lacked the reporter plasmid. Fluorescence was significantly higher in stationary phase cells than in exponentially growing cells, and was stronger for the *E. coli* strain and lowest for the *Alcanivorax* strain, the *Marinobacter* and *Thalassolituus* strains showing intermediate backgrounds levels. This problem was to a large part due to the accumulation of fluorescent compounds in the growth medium, and could be significantly reduced by spinning down the cells and re-suspending them in fresh medium before the biosensor assays.

The *E. coli* reporter showed a rapid, strong response to C_6_-C_10_ alkanes provided that the assay was performed in a mineral salt medium of moderate ionic strength, such as M9. When the hydrocarbons were provided in ONR7a medium, which resembles seawater, *E. coli* cells that had not been pre-adapted to seawater were unable to respond. Pre-adaptation of *E. coli* to the high ionic strength ONR7a medium, which should trigger an osmotic stress response (Hengge-Aronis, 1996), allowed cells to respond to alkanes, although the signal was significantly weaker and less reproducible than when the M9 medium was used. This sensitivity of *E. coli*-based biosensors to seawater has been observed previously, and could be solved by diluting the seawater samples by at least fourfold (Tecon *et al*., 2010). Under optimal conditions – cells grown and assays performed in M9 medium – induction of fluorescence in the *E. coli* biosensor was very good, reaching values about 25-fold higher than the background after 2 h when C_6_, C_8_ or C_10_ alkanes were provided at concentrations above their solubility limits. The *Marinobacter*, *Alcanivorax* and *Thalassolituus* reporter strains, all specialized alkane degraders, provided slower and less intense responses than *E. coli*, although the normalized fluorescence detected after 2–4 h was high. Nevertheless, since the *Marinobacter* and *Thalassolituus* strains showed high background fluorescence levels in the absence of alkanes, the final induction ratios after 2–4 h were in the range of three to eightfold. In part due to its low background fluorescence, the *Alcanivorax* strain showed very high induction ratios, with values of 12 to 14-fold after 2 h, and of up to 50-fold (for C_6_), 23-fold (for C_8_) and 16-fold (for C_10_) after 4 h. These very high values make this strain a very good host for a whole-cell biosensor designed to detect C_6_-C_10_ *n*-alkanes in seawater samples. In this same line, *A. borkumensis* was also found useful to analyse the bioavailability of long-chain alkanes on the basis of a related reporter system sensitive to alkanes of more than 13 carbon atoms (Kumari *et al*., 2011).

Under the experimental conditions used, the lowest concentration of C_8_ to return a clear and statistically significant induction signal was about 0.5 μM for the three marine strains, and 2 μM for the *E. coli* strain. These values are below the solubility limits of C_8_ in water (about 6.3 μM). The linearity of the response was lost at octane concentrations close to and above its solubility limit. Most bacterial reporter assays for hydrocarbons can detect the target compounds in the nanomolar to micromolar range (reviewed in van der Meer *et al*., 2004), the response depending to a large extent on the experimental conditions and the reporter system used.

The usefulness of the reporter strains when confronted with water samples containing commercial petrol or crude oil was also analysed. For the petrol, which contained significant amounts of C_7_-C_9_ *n*-alkanes, the *Alcanivorax* and the *E. coli* strains provided the best results, returning induction values in the range of 25-fold (for *Alcanivorax*) or 14-fold (for *E. coli*) just 2 h after adding petrol at 0.1% (v/v). The *Alcanivorax* strain was clearly superior to *E. coli* in detecting lower petrol concentrations, providing fluorescence induction ratios of sevenfold at petrol concentrations as low as 0.012%, while *E. coli* was barely able to respond at petrol concentrations of 0.025% and rendered no signal at a concentration of 0.012%. The performance of the *Marinobacter* and *Thalassolituus* strains was again compromised by the high background fluorescence observed in the absence of petrol.

The response to crude oil was tested only with the *E. coli* and *Alcanivorax* reporter strains. The crude oil initially used, which was devoid of *n*-alkanes of less than 12 carbon atoms, was essentially unable to induce a response in either strain. Spiking the oil with a mixture of C_6_-C_14_ *n*-alkanes prior to the reporter assay did induce a strong response in both strains (13- to 16-fold). This shows the excellent specificity of the biosensor, which only detects C_6_-C_10_ alkanes; no other oil hydrocarbons induced a signal. Alkanes with between 6 and 10 carbon atoms volatilize rapidly after an oil spill, and their concentration in contaminated seawaters decreases significantly after 2–3 days (Tecon *et al*., 2010). However, they are more toxic than higher molecular weight alkanes due to their greater water solubility, and hence greater bioavailability. Monitoring C_6_-C_10_ alkanes in contaminated water samples is therefore important.

For detecting *n*-alkanes of relatively low molecular weight, both the non-specialized alkane degrader *E. coli* and highly specialized alkane degraders such as *A. borkumensis*, are good hosts for the AlkS/*PalkB-gfp* biosensor derived from the *P. putida* OCT plasmid. However, the usefulness of *E. coli* as a host for this sensing system was compromised by its salt sensitivity and its lower performance when confronted to low hydrocarbon concentrations. *Alcanivorax borkumensis*, a marine bacterium specialized in degrading alkanes, showed threshold detection levels about fourfold lower than those of *E. coli*. In addition, it showed background fluorescence levels in the absence of alkanes significantly lower than those of *E. coli*. Therefore, the better performance of *A. borkumensis* is likely due to a combination of reasons. It is noteworthy that the *Marinobacter* and *Thalassolituus* marine strains also showed fourfold lower detection thresholds for C_8_ than the *E. coli* reporter. The specialization in alkane degradation of the three marine strains may perhaps contribute to their low detection thresholds, possibly because they have evolved efficient uptake systems for these highly hydrophobic molecules. However, little is known about how bacterial cells internalize water-insoluble hydrocarbons. The outer membrane of Gram-negative bacteria is an effective permeability barrier to them. Some strains that can use these compounds as a carbon source are endowed with porins that facilitate, for example, the passage of alkanes (van Beilen *et al*., 2001; Julsing *et al*., 2012; Grant *et al*., 2014; Wang and Shao, 2014) or toluene (Kahng *et al*., 2000; Kasai *et al*., 2001; Hearn *et al*., 2008) through the outer membrane. Thereafter, it is unclear whether they cross the inner membrane using active transport systems or whether they simply diffuse through the hydrophobic lipid bilayer.

The present results highlight the importance of choosing bacterial strains with low background fluorescence values in the absence of an inducer as hosts for biosensor systems based on the GFP. While backgrounds derived from the excretion of fluorescent molecules can be avoided by centrifuging the cells prior to their use in assays, those caused by a high basal expression of the *PalkB-gfp* fusion cannot be easily avoided; a host with low basal expression is therefore vital. Among the strains tested, *A. borkumensis* showed the lowest background fluorescence. In summary, its adaptation to seawater, its low background fluorescence and its superior ability to detect alkanes at low concentrations makes *A. borkumensis* the best host possible – among those tested – for the present reporter system when attempting to detect C_6_-C_10_ alkanes in seawater.

## Experimental procedures

### Bacterial strains and culture media

The strains used in this work were *E. coli* W3110 (Jishage and Ishihama, 1997)*, E. coli* DH5α (Woodcock *et al*., 1989)*, E. coli* HB101 (pRK600) (Kessler *et al*., 1992), *M. hydrocarbonoclasticus* VT8 (Gauthier *et al*., 1992), *A. borkumensis* SK2 (Yakimov *et al*., 1998), *T. oleivorans* Mil-1 (Yakimov *et al*., 2004), *O. messinensis* ME102 (Golyshin *et al*., 2002) and *Cycloclasticus sp*. ME7 (a Mediterranean variant of *C. pugetii*) (Dyksterhouse *et al*., 1995). Unless otherwise stated, *E. coli* strains were grown at 37°C in complete LB medium, or in M9 mineral salts medium supplemented with 0.4% (w/v) glucose and 0.1% (w/v) thiamine (Sambrook and Russell, 2001). The marine strains were cultivated in the artificial seawater mineral salts medium ONR7a, which is based on the ionic composition of seawater (Dyksterhouse *et al*., 1995). *Marinobacter* and *Alcanivorax* strains were cultivated at 30°C using 1% (w/v) pyruvate or 2.5% (w/v) tetradecane as the carbon source. *Thalassolituus* and *Oleiphilus* strains were cultivated at 25°C using 2.5% (v/v) tetradecane as the carbon source; where indicated, 1% acetate or 1% Tween-20, respectively, were used instead. *Cycloclasticus* strains were cultivated at 25°C using 0.25% (v/v) naphthalene as the carbon source.

### Determination of sensitivity to streptomycin

To determine the sensitivity of the hydrocarbonoclastic marine strains to streptomycin (the antibiotic used to select for plasmid pKSB1), cells were cultivated to stationary phase in ONR7a medium with the appropriate carbon source (see Table 1), centrifuged and re-suspended in the same culture medium to a turbidity (A_600_) of 0.1. One hundred μl of each culture were plated onto an ONR7a medium agar plate containing the carbon source of choice, and the cells allowed to adsorb onto the plate for 30 min at room temperature. A streptomycin e-test strip (0.064 to 1024 μg/ml; Biomerieux, France) was equilibrated for 30 min at room temperature and then applied onto the agar surface. Cells were allowed to grow until a lawn was visible. The minimum inhibitory concentration (MIC) was considered to be the point on the strip scale where growth inhibition began to be evident. Tests were performed in triplicate. Using the values obtained as a reference, the assays were repeated in test tubes containing liquid medium. See Table 1 for results.

### Construction of plasmid *pKSB1*

A DNA fragment containing the *alkS* gene (including its own promoter) coding for the alkane-responsive AlkS transcriptional regulator was excised from plasmid pTS1 (Yuste *et al*., 1998) with HindIII and BsaAI restriction endonucleases, and inserted between the HindIII and SmaI sites of plasmid pSEVA 424 (Silva-Rocha *et al*., 2013) to render plasmid pKSAlkS. In parallel, a DNA segment was constructed in which the *gfp* gene is transcribed from promoter *PalkB*, i.e. the AlkS-regulated promoter for the genes of the alkane degradation pathway encoded in the *P. putida* OCT plasmid (Kok *et al*., 1989; Panke *et al*., 1999; Canosa *et al*., 2000). The *gfp* gene was excised from pGreenTIR (Miller and Lindow, 1997) as an EcoRI DNA fragment and the ends blunted with T4 DNA polymerase before cloning it into the KpnI site (blunted with T4-DNA polymerase) of plasmid pPB7 (Yuste *et al*., 1998) under the influence of promoter *PalkB*. The plasmid obtained was named pPBG1. The *PalkB-gfp* transcriptional fusion was excised from pPBG1 with HindIII and SacI, and cloned between the same restriction sites of pUC18Not, yielding plasmid pPBG2. The *PalkB-gfp* fusion was extracted from pPBG2 with NotI and cloned at the HindIII site of pKSAlkS, after blunting the ends of both fragments with T4 DNA polymerase. A plasmid was selected in which the *alkS* gene and the *PalkB-gfp* fusion were in the opposite orientation (named pKSC1). Since this plasmid was found to be unable to replicate in the marine strains used, a DNA fragment including *alkS* and *PalkB-gfp* was excised from it using NotI sites that flanked the DNA fragment (sites that derive from the plasmid vector used), and the DNA segment obtained was cloned into the NotI site of the broad-host-range plasmid pSEVA 431 (Silva-Rocha *et al*., 2013), obtaining plasmid pKSB1.

### Conjugation assays

Plasmid pKSB1 was introduced into the *Marinobacter*, *Alcanivorax, Thalassolituus* and *Oleiphilus* strains by tripartite conjugation assays, using *E. coli* DH5α(pKSB1) as the donor, *E. coli* HB101(pRK600) as a helper of transfer functions and the selected marine strain as the recipient. To this end, *E. coli* strains DH5α(pKSB1) and HB101(pRK600) were cultivated to stationary phase in LB medium supplemented with 50 μg/ml streptomycin or 50 μg/ml of chloramphenicol, respectively, while the marine strains were cultivated to stationary phase in ONR7a medium with the appropriate carbon source. Cells were centrifuged and re-suspended in 1 ml of cLB (10 g/l of triptone, 5 g/l of yeast extract, 0.45 g/l Na_2_HPO_4_ 2H_2_O, 2.5 g/l NaNO_3_, 11.5 g/l NaCl, 0.38 g/l KCl, 0.7 g/l CaCl_2_ H_2_O and 2% sodium pyruvate; Sabirova *et al*., 2006) in donor/helper/recipient proportions of 1:2:4. Cells were placed onto a nitrocellulose filter (0.45 μm pore size) and incubated at 25°C for 24 h. They were then collected from the filter in 1 ml of ONR7a, diluted and plated onto ONR7a agar plates with the required carbon source (C_16_ for *Marinobacter* and C_14_ for *Alcanivorax, Thalassolituus* and *Oleiphilus*, in all cases applied to the plate lid to saturate the vapour phase) and streptomycin (100 μg/ml for *Marinobacter*, 50 μg/ml for *Alcanivorax*, 5 μg/ml for *Thalassolituus* and 2.5 μg/ml for *Oleiphilus*). Dicyclopropylketone (a gratuitous inducer that mimics the effect of alkanes; Grund *et al*., 1975) was also included in the agar plate at a final concentration of 0.05% (v/v) to induce the *PalkB* promoter and allow the expression of the *gfp* gene. Plates were incubated at 30°C for *Marinobacter* and *Alcanivorax* strains, and at 25°C for *Thalassolituus* and *Oleiphilus* strains, until colonies appeared. Colonies showing fluorescence derived from GFP were selected using a luminometer and the presence of plasmid pKSB1 verified by colony polymerase chain reaction (PCR) using the oligonucleotides GFP 300 (5′-AAAGATGACGGGAACTAAAGA-3′) and GFP Rev (5′-GTGAGTTATAGTTGTATTCC-3′), which amplify the *gfp* gene. The phylogenetic identity of the selected transconjugants was checked by PCR amplification and sequencing of the 16S rRNA gene, using oligonucleotides 16S-8a27D (5′-AGAGTTTGATCCTGGCTCAG-3′) and 16S-1510R (5′-GGTTACCTTGTTACGACTT-3′) as primers.

### Electroporation

Plasmid pKSB1 could not be introduced into *Cycloclasticus sp*. ME7 by conjugation; an electroporation protocol was therefore used instead. Bacteria were plated onto ONR7a with naphthalene and, when colonies were evident, cells were taken with an inoculating loop and re-suspended in 250 μl of sterile cold water with 20% glycerol. These cells were then centrifuged and re-suspended in 30 μl of cold water with 20% glycerol. Two hundred ng of plasmid DNA were added, and the mixture was kept on ice for 1 min. It was transferred to an electroporation cuvette (0.1 cm) and a pulse of 1.8 Kv applied in a Micropulser (Biorad). One millilitre of ONR7a was immediately added to the 30 μl, and the mixture transferred to a flask containing 4 ml of ONR7a and 0.25% (w/v) naphthalene as a carbon source. The flask was incubated 4 h at 25°C with agitation. The cells were then plated onto ONR7a plates with 30 μg/ml of streptomycin and naphthalene (provided as vapour from crystals placed on the plate lid) and incubated at 25°C until colonies appeared. The presence of the plasmid was confirmed by PCR using oligonucleotides for the *gfp* gene.

### Fluorescence assays with pure alkanes

A culture of each reporter strain was cultivated to stationary phase in the appropriate medium as specified above (see *Bacterial strains and culture media*). Cells were centrifuged and re-suspended in fresh medium with the appropriate concentration of streptomycin to select for plasmid pKSB1. The carbon source added was glucose for *E. coli*, pyruvate for *Alcanivorax*, acetate for *Thalassolituus*, Tween-20 for *Oleiphilus* and naphthalene for *Cycloclasticus*. For the *Marinobacter* strain, the assays were performed without a carbon source to minimize autofluorescence and improve the signal/noise ratio in the presence of alkanes. After adjusting the turbidity of the culture to 0.9–1 with fresh medium, 2 ml of the cell suspension were loaded into 4.5 ml crew-cap glass vials (one vial per test). In parallel, different alkanes were added to 2 ml of cell-free culture medium; when hydrocarbons were added at 1%, these were directly supplied to the medium and the vials agitated for 1 min to homogenize the mixture and saturate the water phase with the hydrocarbon. When analysing the concentration-dependent response of the reporter cells, alkanes were added from stock solutions prepared in dimethyl sulphoxide. Two millilitres of the alkane-containing medium, or of alkane-free medium for control reactions, were added to the vial containing 2 ml of the cell suspension; the vials were tightly closed to avoid evaporation of the hydrocarbons and incubated at 30°C with agitation (end-over-end) for 1 to 4 h, as specified. Vials containing growth medium with different inducers, but lacking cells, were used as blanks. For measuring fluorescence, 200 μl samples were taken (in triplicate; technical replicates) from the vial and dispensed onto a black, clear-bottomed microtitre plate. The fluorescence (excitation 480 nm, emission 520 nm) and absorbance (600 nm) were measured simultaneously and the values represented as normalized fluorescence, dividing the fluorescence by the absorbance. The fluorescence induction ratio was calculated as the normalized fluorescence of alkane-induced samples over that of non-treated samples. In all cases, three biological replicates were performed.

### Fluorescence assays with petrol and crude oil

Petrol (95 RON) was obtained from a commercial filling station. Dansk blend crude oil, which is devoid of the lighter alkanes, was provided by Danish Underground Consortium. Where indicated, the crude oil was spiked with a mixture of C_6_, C_7_, C_8_, C_9_, C_10_, C_12_ and C_14_ alkanes to reach a final concentration of 7% each (v/v). Reporter assays were conducted essentially as indicated above for pure alkanes, directly adding the petrol or the crude oil to 4.5 ml screw-cap glass vials containing 2 ml of cell-free culture medium and vigorously vortexing before the addition of 2 ml of culture containing the reporter strain. Petrol was added at a final concentration of 0.1%, 0.05%, 0.025% or 0.0125% (v/v). Crude oil was added to reach a final concentration of 0.1% (v/v).

### Analysis of petrol and crude oil by gas chromatography/mass spectrometry

The petrol sample was analysed directly by gas chromatography/mass spectrometry (GC-MS) as indicated below. In the case of the Dansk blend, 0.5 g of oil was extracted with 5 ml of dichloromethane and vortexed for 45 s. One gram of sodium sulphate was added to dry the sample and, after removing the sodium sulphate by centrifugation, the sample was stored at −20°C. Samples were then analysed by GC-MS.

For GC-MS analyses, samples were diluted 100-fold and 1 μl of the diluted sample injected into a GC-MS analyser (model Varian 3800) equipped with a Factor VI column (30 m × 0.25 μm × 0.25 mm). Linear alkanes were identified by their retention time and mass spectra, using pure alkanes as standards prepared at 1000 ppm in dichloromethane. Gas chromatography/mass spectrometry analyses were performed at the Research Interdepartmental Service of the Autonomous University of Madrid, Madrid, Spain.

## Conflict of interest

None declared.
